# Accessibility, Cost, and Quality of an Online Regular Follow-Up Visit Service at an Internet Hospital in China: Mixed Methods Study

**DOI:** 10.2196/54902

**Published:** 2024-10-21

**Authors:** Kun Wang, Wenxin Zou, Yingsi Lai, Chun Hao, Ning Liu, Xiang Ling, Xiaohan Liu, Ting Liu, Xin Yang, Chenxi Zu, Shaolong Wu

**Affiliations:** 1 Department of Medical Statistics School of Public Health Sun Yat-Sen University Guangzhou China; 2 School of Government Sun Yat-sen University Guangzhou China; 3 Sun Yat-sen Global Health Institute Institute of State Governance Sun Yat-sen University Guangzhou China; 4 School of Management Lanzhou University Lanzhou China; 5 China Research Center for Government Performance-Management Lanzhou University Lanzhou China; 6 Third Affiliated Hospital of Sun Yat-sen University Guangzhou China; 7 Department of Health Policy and Management School of Public Health Sun Yat-sen University Guangzhou China

**Keywords:** internet hospital, medical service, accessibility, cost, quality, regular follow-up

## Abstract

**Background:**

Telemedicine provides remote health care services to overcome constraints of time and space in accessing medical care. Similarly, internet hospitals in China support and provide remote health care services. Due to the COVID-19 pandemic, there has been a proliferation of internet hospitals. Many new services, including online consultations and regular online follow-up visit services, can now be accessed via internet hospitals in China. However, the accessibility, cost, and quality advantages of regular online follow-up visit services remain unclear.

**Objective:**

This study aimed to evaluate the accessibility, costs, and quality of an online regular follow-up visit service provided by an internet hospital in China. By analyzing the accessibility, costs, and quality of this service from the supply and demand sides, we can summarize the practical and theoretical experiences.

**Methods:**

A mixed methods study was conducted using clinical records from 18,473 patients receiving 39,239 online regular follow-up visit services at an internet hospital in 2021, as well as interviews with 7 physicians, 2 head nurses, and 3 administrative staff members. The quantitative analysis examined patient demographics, diagnoses, prescriptions, geographic distribution, physician characteristics, accessibility (travel time and costs), and service hours. The qualitative analysis elucidated physician perspectives on ensuring the quality of online health care.

**Results:**

Patients were predominantly middle-aged men with chronic diseases like viral hepatitis who were located near the hospital. The vast majority were from Guangdong province where the hospital is based, especially concentrated in Guangzhou city. The online regular follow-up visit service reduced travel time by 1 hour to 9 hours and costs by ¥6 to ¥991 (US $0.86-$141.32) depending on proximity, with greater savings for patients farther from the hospital. Consultation times were roughly equivalent between online and in-person visits. Physicians provided most online services during lunch breaks (12 PM to 2 PM) or after work hours (7 PM to 11 PM), indicating increased workload. The top departments providing online regular follow-up visit services were Infectious Diseases, Rheumatology, and Dermatology. The most commonly prescribed medications aligned with the prevalent chronic diagnoses. To ensure quality, physicians conducted initial in-person consultations to fully evaluate patients before allowing online regular follow-up visits, during which they communicated with patients to assess conditions and determine if in-person care was warranted. They also periodically reminded patients to come in person for more comprehensive evaluations. However, they acknowledged online visits cannot fully replace face-to-face care.

**Conclusions:**

Telemedicine services such as online regular follow-up visit services provided by internet hospitals must strictly adhere to fundamental medical principles of diagnosis, prescription, and treatment. For patients with chronic diseases, online regular follow-up visit services improve accessibility and reduce cost but cannot fully replace in-person evaluations. Physicians leverage various strategies to ensure the quality of online care.

## Introduction

### Significance of Online Health Care

Time and space are the basic environments for human survival and development. One of the goals of human development is to break through the shackles of time and space. In the health care sector, time and space have always constrained both the supply and demand sides in accessing and providing health services, known as spatial accessibility and temporal accessibility. Therefore, how can we improve the spatial and temporal accessibility of health care services? Telemedicine actually provides one viable approach. Telemedicine refers to the use of electronic and telecommunication technologies to deliver health care services remotely [1]. It has been applied in health care since the invention of the telegraph and telephone. Passing through successive evolution phases involving radio, television, the internet, mobile health, and artificial intelligence, telemedicine has now become an integral component of health care delivery [2].

Initially, telemedicine only enabled the transmission of text and voice using telegraphy and telephony. Later, television technology allowed the transmission of medical images. With the popularization of digital technology and the internet, multimedia medical information could be inexpensively transmitted to the masses. Especially with the proliferation of smartphones, almost everyone can now access health information services anytime, anywhere [2]. With the addition of artificial intelligence, 5G networks, virtual reality, and wearable technology, telemedicine appears almost omnipotent [3-6]. As telemedicine has advanced, more traditional health care services have shifted from offline to online. Its applications have expanded from basic consultations to even complex remote surgeries [7].

Instant online health care services offer a significant advantage in terms of spatial accessibility as they eliminate the need for face-to-face interactions between health care service providers and recipients. Generally, online health care services are also more cost-effective than offline services. However, in comparison with offline services, the quality of online health care services tends to be relatively lower [8]. Due to concerns related to quality and risks, health care professionals and institutions are often hesitant to provide core health care services online. Additionally, government regulations may restrict the provision of certain services, such as initial diagnosis, through online platforms [9]. Therefore, what types of services can be offered online? How substantial are their advantages in terms of accessibility and cost? How can the quality of health care services be ensured?

Existing research on health service delivery shows that telemedicine delivered through the internet can provide appointment scheduling, consultation, payment, examination, report checking, symptom monitoring, prescriptions, medical conferences, follow-up visits, and even simple surgeries online [7,10,11]. For the most essential aspects of health care services—diagnosis, prescription, and treatment—these online services are only applicable to specific situations, such as mild symptoms, certain diseases (eg, skin and mental diseases), follow-up visits, remote areas lacking vessels and aircraft, and other medical conditions suitable for online health care. In terms of large-scale application, telemedicine is mostly used for regular follow-up visit services. A “regular online follow-up visit” refers to a specific service provided by internet hospitals for patients who require regular follow-up visit services. Doctors prescribe medications online for stable follow-up patients and have the medications delivered to them. How much time and distance can online regular follow-up visit services save in terms of accessibility? How much can patients in different regions save in terms of expenses? How can the quality of online regular follow-up visit services be ensured? Through a mixed methods study with China’s internet hospital, this paper attempted to answer these questions. On this basis, the paper further discusses the impact of telemedicine on health care service delivery.

### Online Health Care in China

In China, medical resource distribution is unbalanced, with large urban-rural and regional differences [12,13]. Most high-quality medical resources are concentrated in the city's top tertiary public hospital [11,14]. In addition, a sound system of tiered diagnosis and treatment has not yet been established. Patients can choose medical institutions freely. In order to obtain high-quality medical services, patients flock to the tertiary hospitals [15]. As a result, tertiary public hospitals are crowded, but primary health care institutions are deserted. Through internet-based online consultation services, patients in remote and rural areas can conveniently access the services of physicians from tertiary hospitals through an internet hospital. Internet hospitals are a platform to provide remote health care services [16]. Internet hospitals have greatly improved the accessibility of high-quality medical services from tertiary hospitals, especially those in Beijing, Shanghai, and Guangzhou.

After the internet entered China in the 1990s, a variety of health-related websites also emerged. In the beginning, most websites only provided services such as health counseling or drug searches. With the advancement of technology, some internet platforms began to provide non-core medical services such as registration, payment, the purchase of medicines, and chronic disease management [8]. Some internet health care companies have begun to penetrate into the core areas of health care by offering medical consultations called *light consultations*. Apart from not providing prescriptions or treatment plans in compliance with regulations, there is no clear boundary between a light consultation and medical diagnosis. Eventually, the internet hospital established by the Guangdong Second Provincial General Hospital made a breakthrough. It carried out online video-based diagnosis and treatment through an internet platform. After issuing electronic prescriptions, patients could obtain medications at partner pharmacies or primary care facilities [17]. According to the different initiators, China's internet hospitals can be divided into 3 categories: government-led internet hospitals, public hospital–led internet hospitals, and enterprise-led internet hospitals [16]. Our study mainly focused on an internet hospital led by a public hospital.

Prior to 2018, internet health care companies and hospitals were actively exploring internet hospitals, but internet hospitals were still in the initial stages of development. Policies for internet hospitals were also not yet mature. In 2018, the General Office of the State Council issued an opinion on Promoting the Development of Internet plus health care in order to encourage the development of internet hospitals [18]. In order to implement the policy, the National Health Commission and the State Administration of Traditional Chinese Medicine organized and formulated the Internet Diagnosis and Treatment Management Measures (trial implementation), Internet Hospital Management Measures (trial implementation), and Telemedicine Service Management Standards (trial implementation) [9]. The issue of these 3 documents was the first time that China put forward detailed regulations on internet hospitals. This was a symbol that internet hospitals had entered the stage of standardized development in China [17].

After the issue of these 3 documents and the COVID-19 pandemic, internet hospitals were rapidly established in China. In 2017, 68 internet hospitals were identified in research [19]. In 2019, there were 158 internet hospitals nationwide [20]. However, in 2021, there were more than 1600 internet hospitals in China [21]. During the initial period of the COVID-19 pandemic, the Chinese government took strict quarantine measures to prevent the spread of the virus. It also prevented citizens from getting face-to-face medical services. Simultaneously, most of the hospitals were used to treat patients who had been infected, and they reduced offline outpatient services [22]. With the control of COVID-19 spread, it was a challenge to provide constant medical services to the large number of patients with chronic diseases [23]. At this point in time, internet hospitals were favored due to their noncontact nature. During the COVID-19 epidemic, internet hospitals not only provided people with health information on COVID-19 but also ensured constant medical services for patients with chronic diseases. Patients with chronic diseases can register for online consultations on internet hospitals, and physicians can confirm the patients’ conditions based on their past medical records and online consultations. Medicines are delivered to the patient by a specialized courier company after the prescription is issued [24]. During the pandemic, the government leveraged internet hospitals to ensure patients without COVID-19 could still access medical services while alleviating the burden on health care facilities [21,25,26].

Internet hospitals continued to play a role after China lifted a series of blocking policies against the COVID-19 pandemic. Internet hospitals break down the barriers of time and space and improve the accessibility of health services [15]. During the COVID-19 pandemic, people used internet hospitals due to the lack of direct contact between physicians and patients (ie, high accessibility). However, in the later stages of the pandemic, the quality of online health care services became an issue of greater concern. The advantage of the accessibility of online health care services is undisputed, but the services’ quality has been under discussion [27-29]. In order to ensure the medical quality of online health care services, the Internet Diagnosis and Treatment Management Measures (trial implementation) clearly states that physicians can issue online prescriptions for some common diseases and chronic diseases only if they clearly understand the patient's medical record information [9]. Online regular follow-up visit services in public hospitals are conducted under this law. This study aimed to analyze the advantages and disadvantages of a particular service delivered by online hospitals—regular follow-up visit services—from the perspective of accessibility, cost, and quality. By analyzing the accessibility, cost, and quality of online regular follow-up visit services from the supply and demand sides, we can summarize the practical and theoretical experiences.

## Methods

### Conceptual Framework

**Figure 1:**
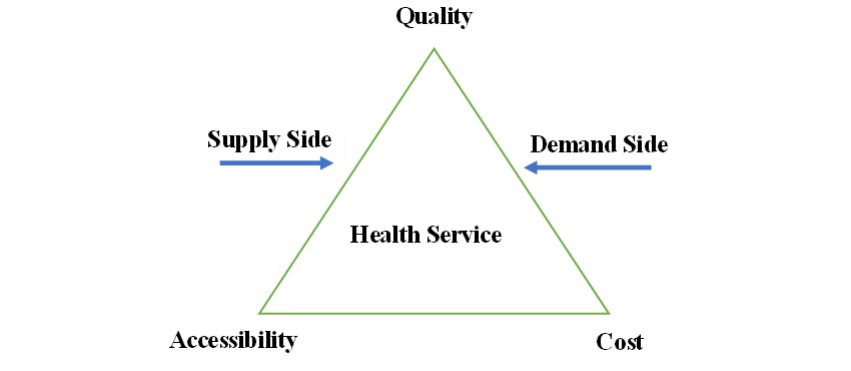
The health service triangle model.

There are 2 very famous models of health care service delivery: the World Health Organization (WHO) health system model focusing on the supply side and the model by Andersen and Davidson [30,31] concentrating on the demand side. In 2000, the WHO published a report that established a milestone health system framework [32]. The health system includes 4 functions—stewardship, creating resources, financing, and delivering services—that determine 3 goals: health, responsiveness, and fairness. Using this model, the WHO evaluated the health system performance of its 192 member states. In 2010, 4 intermediate goals—accessibility, coverage, quality, and safety—were added to the model in order to link functions and objectives [30]. The model by Andersen and Davidson, since its establishment in the 1960s, has evolved several times into a series of health service frameworks. The latest model by Andersen and Davidson consists of 4 components—environment, population characteristics, health behavior, and outcomes—elaborating the interrelationships between health care service accessibility, utilization, and satisfaction [31]. Specifically, satisfaction includes quality, financing, convenience, and availability. The model by Andersen and Davidson has been widely used in studies on the equity of health care service accessibility and utilization. In both models, health services as the most essential element connect the supply and demand sides, aiming to promote, restore, and maintain health. The accessibility, quality, and cost of health services play critical roles in both models as key indispensable components. Accordingly, we established a health service triangle model covering both supply and demand sides, as shown in Figure 1, to analyze the impacts of telemedicine on the accessibility, quality, and cost of online regular follow-up visit services in China.

### Data

#### Patient Care Access

Prior to receiving health care services, patients need to register either onsite at the hospital or online. Patients see a physician face to face within a week after they register online. However, via the internet hospital established by the Third Affiliated Hospital of Sun Yat-sen University (TAHSYSU), patients only need to log on to the TAHSYSU app on their cell phone and input their personal information, medical examination report, and previous prescriptions on the regular follow-up visit service page. Within 24 hours, the attending physician at TAHSYSU confirms their identity information and issues the appropriate prescription according to the patient’s condition.

#### Demand Side

Data were collected for patients who received online regular follow-up visit services from TAHSYSU in 2021. The data included 64,202 drug records and 39,239 medical prescriptions for 18,473 patients who received the online regular follow-up visit service. The data provided 3 kinds of information. The first was general information about patients, including age and gender. The second was the address information where the patient will receive the medication delivery. The third was the department from which patients received online medical services, their disease, when a medical prescription was created, drug names, and the number of drugs.

#### Supply Side

Data were collected for physicians who provided online medical consultation and online regular follow-up visit services in the TAHSYSU from the WeChat mini program called TAHSYSU Internet Hospital. On the register page, the internet hospital departments are shown. If a patient selected a physician based on their condition, the physician’s information, including their technical title and total number of service occurrences that the physician has provided, were shown. We also collected data about health care service accessibility, quality, and cost from physicians, nurses, and administrators through interviews.

### Quantitative Methods

The features of the patients were described first. The patient address information was parsed into 3 levels of administrative regions, including district, city, and province (or municipality), as well as specific latitudes and longitudes, by calling the Amap web service application programming interface (API) using Python programs. The geographical distributions of patients at the district, city, province, and region levels were described. The characteristics of the patients were then described, including age, gender, departments in which patients received online medical services, diseases, and kinds of drugs.

Quantitative methods were mainly used to measure the advantages and disadvantages of online health care services in terms of cost and accessibility. The data to measure costs and accessibility came from map software, rather than patient surveys. A Python program was written to access the Amap web service API for driving route planning and public transportation route planning to estimate the time and cost for patients to travel to face-to-face visits. We used 9 AM to 11 AM and 2 PM to 4 PM as the simulation periods for patients' visits to the hospital. The time and cost for patients to travel to the hospital by public transportation or by car were simulated. The average of the simulation outcomes was used as the estimation result. The time and cost of drug delivery were obtained by checking the official website of SF Express. By comparing the time and costs, the advantages and disadvantages of offline and online regular follow-up visit services can be observed.

Finally, the features of physicians were described. The frequency of services provided by physicians in different departments and with different professional titles was described. By using the timing of medical prescription creation from the patient data, we could determine the number of prescriptions prescribed by physicians during different time periods within a day in order to analyze the accessibility of prescriptions.

### Qualitative Methods

Qualitative methods were mainly used to discuss the advantages and disadvantages of online health care services in terms of medical quality. Semistructured interviews were conducted at TAHSYSU based on the descriptive statistics of the physicians. The interviews were designed to mainly answer 5 broad questions posed to physicians: (1) How can medical service quality be ensured during the online regular follow-up visit service process? (2) Which diseases and conditions would you recommend for patients to use the online regular follow-up visit service? (3) Is there any difference between the types of drugs prescribed online and those prescribed offline? (4) What other perspectives do you have on the quality of health care services provided by online hospitals? (5) What other thoughts do you have about the online regular follow-up visit service? For other interviewees, we asked them about their thoughts about internet hospitals. Interviews lasted approximately 30 minutes per interviewee. We stopped inviting new interviewees when the interviews stopped generating new information. Ultimately, 12 interviewees were selected from different departments in the hospitals. Of the 12 interviewees, there were 7 physicians, 2 head nurses, and 3 administrative staff members. Of the interviewees, 7 were physicians who regularly used such services from departments with high internet hospital utilization. After the interview, the audio data were catalogued and transcribed verbatim by a third-party company specializing in transcriptions in the Chinese language. Subsequently, using a grounded theory approach, we coded the text to identify motivations, risks, benefits, disease characteristics, quality control strategies, and medication traits associated with the online provision of health care. Ultimately, these data were used to elucidate how physicians determine patient suitability for online health care services and how they ensure quality of the online health care services provided.

### Ethics Approval

The Human Studies Committee of Third Affiliated Hospital of Sun Yat-sen University (TAHSYSU) approved the study’s protocol in compliance with the Declaration of Helsinki—Ethical Principles for Medical Research Involving Human Subjects (number 2022 02-279-01).

## Results

### Demand Side

#### Demographic Characteristics and Visits

The results are shown by age group and gender in Table 1. In total, 18,473 patients received 39,239 online regular follow-up visit services in 2021. Patients were predominantly 21 years to 40 years old (10,036/18,473, 54.33%) and male (10,538/18,473, 57.05%). The average number of visits per patient was 2.12. Patients aged 41 years to 60 years had the highest average number of visits (2.33), while those older than 80 years had the lowest number of visits (1.75). Male patients had a higher average number of visits (2.27) than female patients (1.93). Overall, middle-aged and male patients contributed to more visits on average.

**Table 1:** Demographic characteristics of patients (N=18,473) and the average number of visits (N=39,239; overall mean: 2.12 visits per person).

Characteristic		Visits, n (%)	Patients, n (%)^a^	Visits per person, mean
**Age (years)**				
	≤20	1078 (2.75)	702 (3.8)	1.54
	21-40	20,566 (52.41)	10,036 (54.33)	2.05
	41-60	14,168 (36.11)	6093 (32.98)	2.33
	61-80	3315 (8.45)	1578 (8.54)	2.10
	≥80	112 (0.29)	64 (0.35)	1.75
**Gender**				
	Female	15,298 (38.99)	7935 (42.95)	1.93
	Male	23,941 (61.01)	10,538 (57.05)	2.27

^a^If 2 patients had the same district and ID information, they were considered the same person.

#### Geographical Distribution

**Figure 2:**
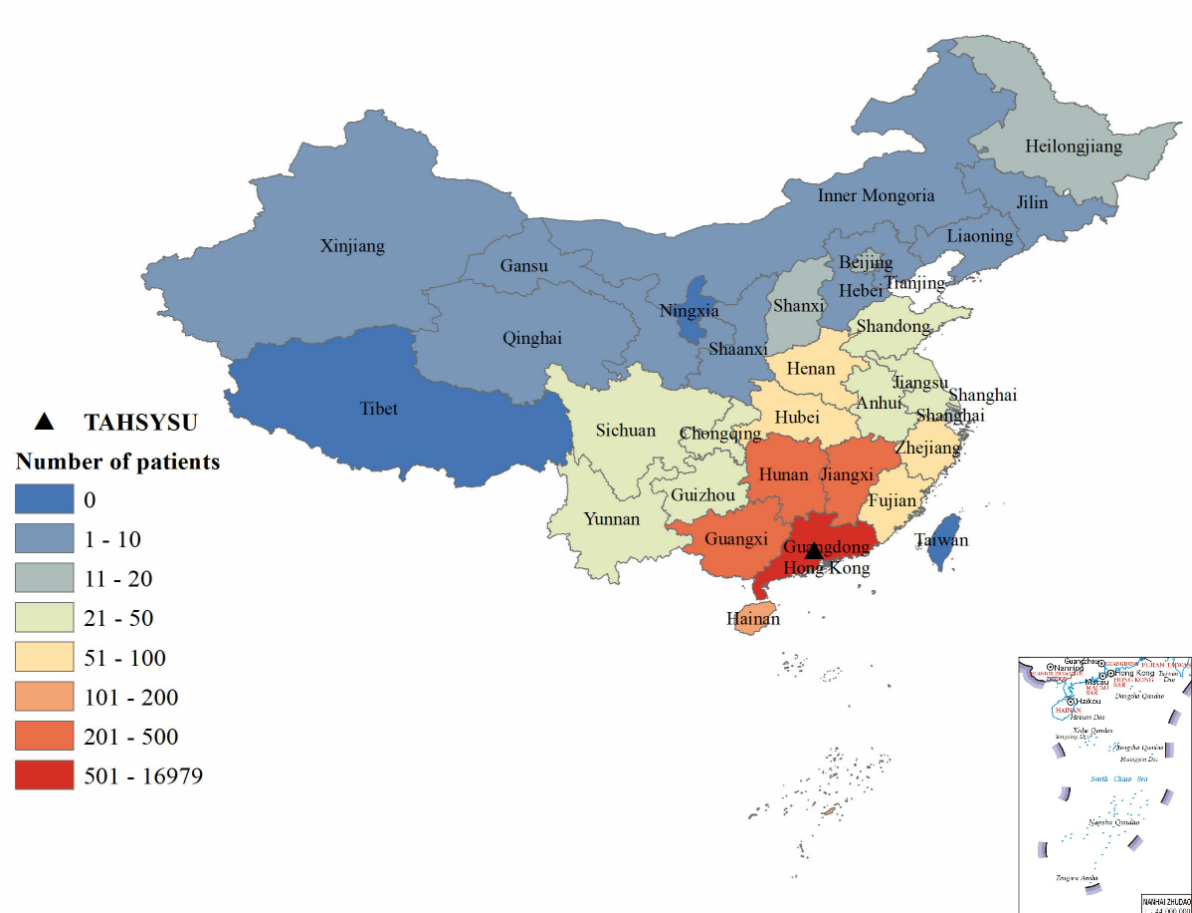
Province-level geographical distribution of patients. TAHSYSU： Third Affiliated Hospital of Sun Yat-sen University.

**Figure 3:**
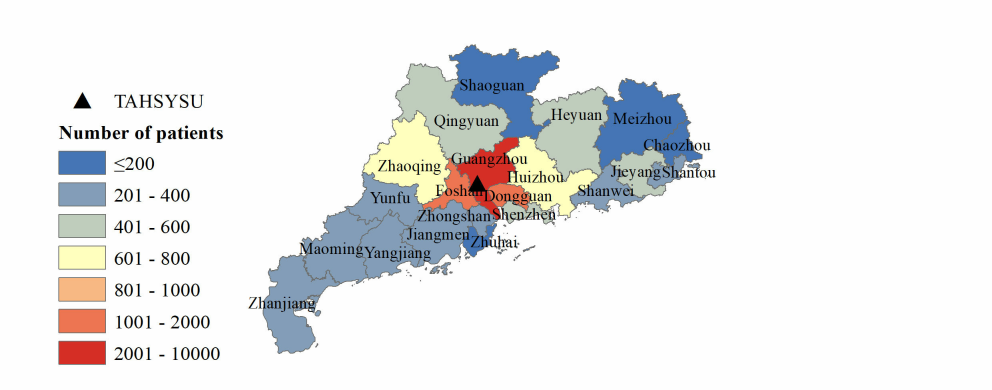
City-level geographical distribution of patients. TAHSYSU: Third Affiliated Hospital of Sun Yat-sen University.

**Figure 4:**
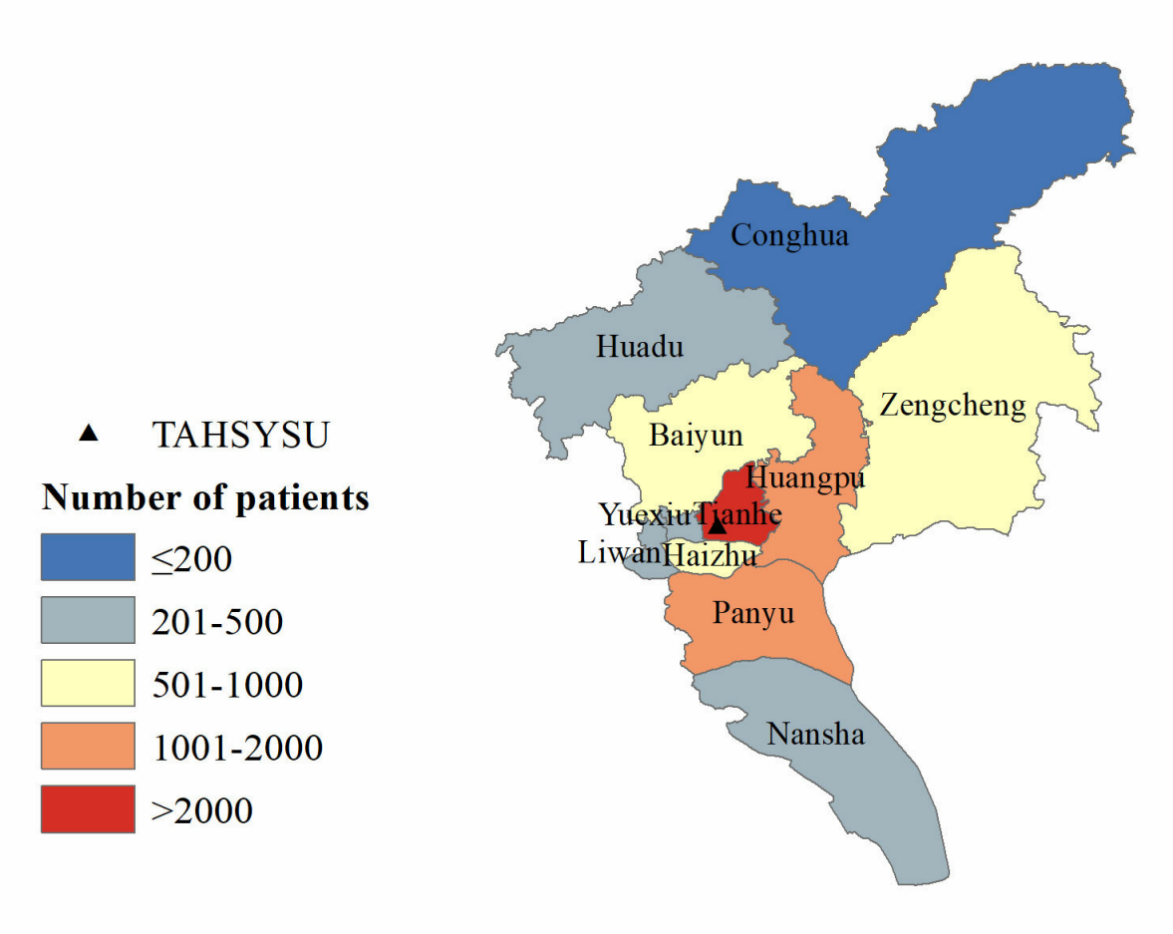
District-level geographical distribution of patients. TAHSYSU: Third Affiliated Hospital of Sun Yat-sen University.

Table 2 shows the geographical distribution of patients and average number of visits by region in China. The vast majority of patients were from south China (17,376/18,473, 94.06%), which also accounted for 94.71% (37,163/39,239) of total visits. Patients from east China comprised 2.37% (437/18,473) of the sample and 2.14% (840/39,239) of visits. The average number of visits per patient was highest in south China (2.14) and lowest in northeast China (1.52). The results show that the patient population was highly concentrated in south China, which drove a higher number of total visits and visits per patient compared with other regions. As shown in Table S1 in Multimedia Appendix 1 and Figure 2, the majority of patients from south China were located in Guangdong province, with 16,979 of the 18,473 patients (91.91%) from Guangdong. As shown in Tables S2 and S3 in Multimedia Appendix 1, Figure 3, and Figure 4, most patients were located in Guangzhou city, concentrated in the Tianhe and Huangpu districts where the hospital is located. Tianhe and Huangpu had the highest number of patients due to proximity to the hospital. Yuexiu district attracts patients from nearby areas—Baiyun, Haizhu, and Liwan districts—due to its concentrated medical resources. In summary, the distribution of patients across provinces nationally and cities in Guangdong appears to follow a pattern of more patients in closer proximity to the hospital; however, the distribution across districts in Guangzhou city does not follow this pattern.

**Table 2:** Region-level geographical distribution of patients (N=18,473) and average number of visits (N=39,239; overall mean: 2.12 visits per person).

Regions of China	Visits, n (%)	Patients, n (%)	Visits per person, mean
South China	37,163 (94.71)	17,376 (94.06)	2.14
East China	840 (2.14)	437 (2.37)	1.92
Central China	795 (2.02)	426 (2.31)	1.87
Southwest China	260 (0.66)	128 (0.69)	2.03
North China	96 (0.24)	55 (0.3)	1.75
Northwest China	47 (0.12)	26 (0.13)	1.81
Northeast China	38 (0.1)	25 (0.13)	1.52

^a^If 2 patients had the same district and ID information, they were considered the same person.

#### Description of Patients’ Characteristics by Frequency of Regular Follow-Up Visits

According to the descriptive results described in the previous section, patients received online regular follow-up visit services on average 2.12 times a year. Therefore, the patients were divided into 3 groups, namely, patients who visited once a year, patients who visited twice a year, and patients who visited ≥3 times a year. As shown in Table 3, nearly one-half (9216/18,473, 49.89%) of the patients received online regular follow-up visit services once a year: the older the patient, the more often he or she received online regular follow-up visit services. There was also a gender difference, with slight differences between men and women in the patients who visited once, and men representing significantly larger proportions of patients who visited twice (2343/4034, 58.08%) or ≥3 times (3417/5223, 65.42%). The proportion of patients who visited once a year was highest in Guangzhou (4427/9216, 48.04%), and as the number of visits increased, the proportion of patients from Guangzhou and other provinces decreased, while the proportion of patients from other cities in Guangdong Province increased.

**Table 3:** Characteristics of patients by frequency of regular follow-up visits.

Characteristics		Visits per year		
		Once (n=9216)	Twice (n=4034)	≥3 times (n=5223)
Age (years), mean (SD)		38.57 (14.51)	40.19 (13.73)	41.61 (12.54)
**Gender, n (%)**				
	Female	4437 (48.14)	1691 (41.92)	1806 (34.58)
	Male	4779 (51.86)	2343 (58.08)	3417 (65.42)
**District, n (%)**				
	Guangzhou city	4427 (48.04)	1635 (40.53)	1942 (37.18)
	Other cities in Guangdong province	3979 (43.17)	2063 (51.14)	2933 (56.16)
	Other provinces	810 (8.79)	336 (8.33)	348 (6.66)
**Departments (top 10), n (%)**				
	Infectious Diseases	4487 (48.69)	2537 (62.89)	3904 (74.75)
	Dermatology	1292 (14.02)	363 (9)	179 (3.43)
	Rheumatology	785 (8.52)	342 (8.48)	329 (6.30)
	Gynecology	508 (5.51)	133 (3.3)	86 (1.65)
	Endocrinology	324 (3.52)	87 (2.16)	93 (1.78)
	Hepatobiliary Surgery	306 (3.32)	133 (3.3)	215 (4.12)
	Cardiovascular Internal Medicine	235 (2.55)	59 (1.46)	70 (1.34)
	Neurology	159 (1.73)	71 (1.76)	89 (1.70)
	Nephrology	141 (1.53)	67 (1.66)	72 (1.38)
	Gastroenterology	132 (1.43)	47 (1.17)	43 (0.82)

In the Infectious Diseases department, the proportions of patients who visited twice or ≥3 times were higher than the proportion of patients who visited once a year. However, the situation was the opposite in the Dermatology department. Therefore, the frequency of online regular follow-up services that patients receive varies among patients with different types of diseases.

### Supply Side

#### Physician Departments

As shown in Table S4 in Multimedia Appendix 1, infectious diseases accounted for the largest percentage of visits (26,163/39,239, 66.68%) and patients (10,935/18,473, 59.2%), with an average of 2.39 visits per patient. Rheumatology and Immunology was the second highest ranked department by visits (2804/39,239, 7.15%) and patients (1442/18,473, 7.81%), with an average of 1.94 visits. Dermatology had the third highest proportion of patients (1828/18,473, 9.9%) but a lower visit rate of 1.45 visits per patient. The departments with the highest average visits per patient were Infectious Diseases (2.39 visits), Hematology (2 visits), and Interventional Radiology (2.22 visits). Anorectal Surgery and Vascular Surgery had the fewest visits and patients overall. It is worth noting that the departments with the highest volume of regular follow-up visit services were the hospital’s signature specialty departments. The Infectious Diseases department at TAHSYSU primarily specializes in the diagnosis and treatment of various forms of viral hepatitis. In summary, the results show a concentration of online regular follow-up visit services in infectious diseases and rheumatology, with a significant variation in visit rates across departments.

#### Online Service Volume

The data in Table 4 reflects activity at the TAHSYSU internet hospital since the start of the online medical services, including online consultation services and online regular follow-up visit services, until February 25, 2023. A total of 315,172 online medical services were provided by 375 physicians. Associate chief physicians provided the most services (139,530/315,173, 44.27%) but accounted for only 41.07% (154/375) of physicians. Chief physicians provided 38.76% (122,146/315,173) of the services but accounted for only 22.40% (84/375) of the physicians, with the highest average number of services provided per physician (1454.12). Attending physicians accounted for 32% (120/375) of physicians but only 16.79% (52,913/315,172) of the services, with an average of 440.94 services per physician. Physicians with other titles accounted for only 0.18% (583/315,172) of the services. In brief, higher-ranked physicians including associate chief and chief physicians provided the majority of services and saw higher service volumes per physician.

**Table 4:** Online service volume (N=315,172) provided by physicians with different technical titles (N=375) from the start of the online medical service until February 25, 2023 (average number of services per physician: 840.46).

Technical titles	Services provided, n (%)	Physicians, n (%)	Services provided per physician, mean
Associate chief physician	139,530 (44.27)	154 (41.07)	906.04
Chief physician	122,146 (38.76)	84 (22.4)	1454.12
Attending physician	52,913 (16.79)	120 (32)	440.94
Other technical titles	583 (0.18)	17 (4.53)	32.29

#### Online Service Hours

**Figure 5:**
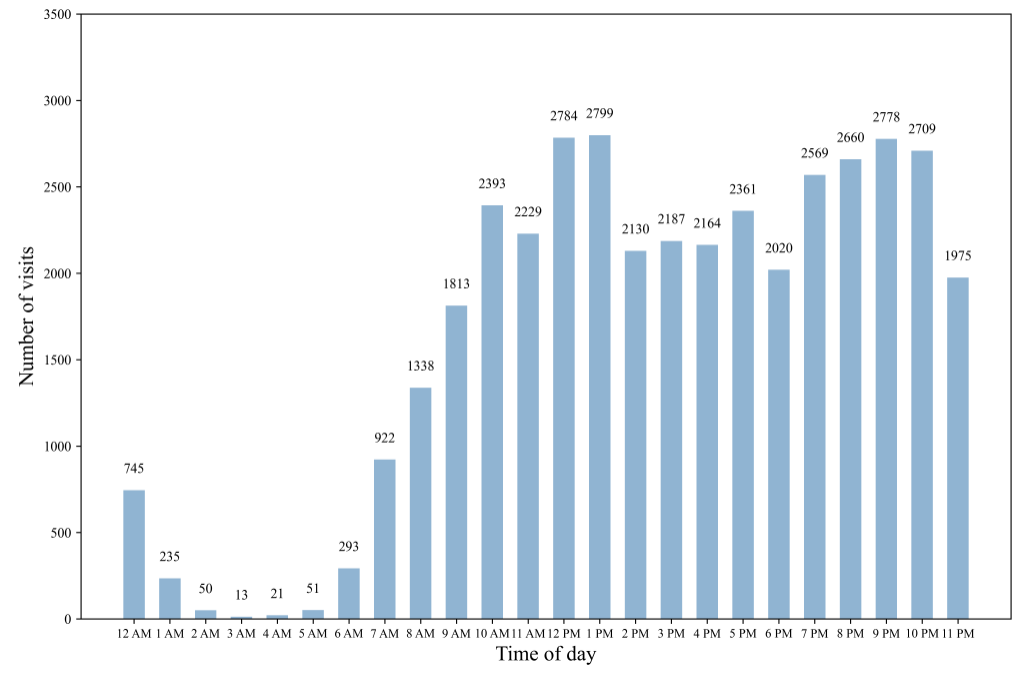
Times of day physicians performed visits with patients online; each time on the x axis indicates a full hour (eg, 1 PM=1 PM to 1:59PM).

As shown in Figure 5, physicians had 2 peak times when they responded to patients via the internet hospital, from 12 PM to 1:59 PM and from 7 PM to 10:59 PM. These 2 time periods are usually freer times for physicians. For instance, 12 PM to 1:59 PM is their lunch break, and 7 PM to 10 PM is their off-duty time. Overall, after the physician had met with the patients who were seen face to face or during his or her own free time, they met with the patients via online regular follow-up visits.

Internet hospital services have subtly increased physicians’ working hours:

Internet hospitals are acting as a triage. Continuity of medical care for patients can be enhanced through online regular follow-up visit services at internet hospitals. But with the increase in the number of patients, we are also struggling. Many patients with stabilized conditions ask a lot of questions during online regular follow-up visit services out of concern for their condition, which increases our workload.

P4, associate chief physician, Endocrinology department

### Health Services

#### Disease Diagnoses

After excluding patients with a diagnosis of counseling and missing records for a diagnosis, 5233 patients had a definite diagnosis. As shown in Table 5, chronic viral hepatitis B was the most common diagnosis, accounting for 49.3% (2580/5233) of the diagnoses. Chronic active viral hepatitis B (491/5233, 9.38%), hepatopathy (305/5233, 5.83%), and liver transplantation status (155/5233, 2.96%) were also among the top diagnoses, indicating a technology advantage for liver-related conditions at TAHSYSU.

**Table 5:** The diseases of patients using online health care services (N=5233; top 10 diagnoses, after removing counseling).

Diagnosis	Patients, n (%)
Chronic viral hepatitis B	2580 (49.30)
Chronic active viral hepatitis B	491 (9.38)
Hepatopathy	305 (5.83)
Liver transplantation status	155 (2.96)
Decompensated liver cirrhosis after viral hepatitis B	118 (2.25)
Acne	118 (2.25)
Systemic lupus erythematosus	80 (1.53)
Malignant tumor of liver	54 (1.03)
Rheumatoid arthritis	54 (1.03)
Chronic nephritic syndrome	50 (0.96)

The top 10 diagnosis were all chronic diseases. This means that patients have a clear diagnosis and a clear treatment plan, which makes the diseases more suitable for online treatment. One of the physicians interviewed said the following:

The most important characteristics of chronic diseases are that they require long-term medication and are not so dangerous. For patients who need to take medication for a long period of time, it is burdensome for them to travel to the pharmacy every time they need to refill their medication. Therefore, the internet hospital's chronic disease follow-up service is developing rapidly.

P1, associate chief physician, Infectious Diseases department

#### Prescribed Drugs

As shown in Table 6, of the 64,202 medications, the most commonly prescribed drugs were tenofovir disoproxil fumarate tablets (10,209/64,202, 15.9%) and entecavir dispersible tablets (9702/64,202, 15.11%), both used to treat chronic hepatitis B. In summary, the main drugs prescribed align with the common diagnoses of liver diseases and their complications.

**Table 6:** Drugs prescribed online by physicians (top 10; N=64,202).

Name of drug	Prescriptions, n (%)
Tenofovir disoproxil fumarate tablets	10,209 (15.9)
Entecavir dispersible tablets	9702 (15.11)
Tenofovir alafenamide fumarate tablets	3724 (5.8)
Isotretinoin soft capsules	1599 (2.49)
Danshnewuweizi tablet	1298 (2.02)
Calcium carbonate and vitamin D3 tablets	828 (1.29)
Methylprednisolone tablets	772 (1.2)
Compound glycyrrhizin tablets	746 (1.16)
Vitamin E soft capsules	734 (1.14)
Hydroxychloroquine sulfate tablets	721 (1.12)

Drugs represent a specialized and sensitive issue. For patients who are followed up at the TAHSYSU internet hospital, the physician prescribes medication based on the patient's past medical history and current condition. However, physicians are very cautious when prescribing medications for first-time patients who visit the internet hospital. One of the physicians interviewed said the following:

I have never prescribed medication to a patient at an internet hospital on a third-party platform such as Haodaifu. But on the TAHSYSU internet platform, sometimes I can't stand the patient's request to prescribe drugs to the first-time patients. Usually, the drugs I prescribe are safer and more conservative, and I will not prescribe drugs with stronger side effects to my patients.

P2, associate chief physician, Cardiovascular department

### Accessibility of Online Services

As shown in Table 7, online medical services save the time patients spend on appointment registration, waiting in the hospital, and traveling. Patients from Guangzhou, other cities in Guangdong, or other provinces in China spend 1.11 hours, 3.66 hours, and 9.35 hours, respectively, traveling by public transportation. If they choose to drive, they spend 0.74 hours, 2.51 hours, and 9.62 hours, respectively. However, if patients see a physician online or face to face, it takes several minutes of the physician’s time to understand the patient’s condition and prescribe medications. Online medical services have no time advantages related to the consultation and prescriptions. After physicians complete the diagnosis and prescriptions, patients using the online medical service receive their drugs by express shipments, and patients using the face-to-face medical service go to hospital pharmacy to get drugs. Getting to the hospital pharmacy costs less time than express delivery. Overall, the online medical service has advantages in appointment registration times, waiting times, and travel times but has no advantages in the time it takes for the consultation, prescriptions, and receiving the drugs. The outstanding advantages in travel time make the disadvantages negligible.

**Table 7:** Comparative estimation of accessibility associated with offline versus online regular follow-up visit services, by location.4

Accessibility features		Guangzhou City	Other cities in Guangdong province	Other provinces
**Appointment registration and waiting times**				
	Face to face (weeks)	≤1	≤1	≤1
	Online (hours)	≤24	≤24	≤24
**Time to hospital (hours), mean (SD)**				
	One-way public transportation	1.11 (0.52)	3.66 (1.38)	9.35 (7.36)
	Drive one way	0.74 (0.29)	2.51 (1.10)	9.62 (5.98)
	Online	0	0	0
**Consultation and prescribing time**				
	Face to face	Several minutes	Several minutes	Several minutes
	Online	Several minutes	Several minutes	Several minutes
**Time for patient to receive drugs (hours)**				
	Hospital pharmacy	≤1	≤1	≤1
	Express delivery, mean (SD)	27.00 (0.62)	28.78 (5.69)	48.22 (17.00)

### Cost of Online Services

Table 8 shows the characteristics of online health care services, such as the savings in indirect medical costs associated with the fact that patients do not need to travel to a hospital. No matter how far away the patient lives, the online health care service costs ¥0 for travel. Patients from Guangzhou, other cities in Guangdong, or other provinces in China spend ¥5.83, ¥98.38, and ¥359.41, respectively, to travel by public transportation to the hospital. If they choose to drive, they spend ¥21.14, ¥208.34, and ¥990.77, respectively. There is a standardized consultation fee of ¥10 for the online regular follow-up visit service. If you choose to have a face-to-face consultation, it costs at least ¥10 for the consultation, depending on the technical title of the physician you choose. Patients pay express delivery fees for online medical services but not for in-person hospital visits. Overall, online medical services save a lot of money for patients, especially patients located far from the hospital.

**Table 8:** Comparative estimation of costs associated with offline versus online regular follow-up visit services, by location.

Costs			Guangzhou City		Other cities in Guangdong province		Other provinces
**Fees to get to the hospital (¥), mean (SD)**							
	One-way public transportation	5.83 (3.24)		98.38 (84.38)		359.41 (244.25)	
	Drive one way	21.14 (18.00)		208.34 (147.03)		990.77 (650.53)	
	Online	0		0		0	
**Consulting and prescribing** **fees (¥)**							
	Face to face	≥10		≥10		≥10	
	Online	10		10		10	
**Fees for drug prescriptions (¥), mean (SD)**							
	Hospital pharmacy	0		0		0	
	Express delivery (≤1 kg)	12.00 (0)		13.00 (0)		18.04 (0.34)	

### Quality of Online Services

According to the aforementioned examination of diseases and pharmaceuticals, the cohort using online regular follow-up visit services seems to be largely comprised of patients with chronic pathologies or those in the chronic stage of their diseases. Chronic pathologies are typified by discrete diagnostic and treatment paradigms. For patients with chronic diseases receiving standardized therapeutic interventions, their morbidity seems unlikely to exhibit precipitous, perilous perturbations in the short term, and their pharmaceutical regimen also does not have considerable variability. In accordance with guidelines, such characteristics appear conducive to return online visit services provided by the internet hospital.

I will decide whether the patient will have an online follow-up or a face-to-face follow-up based on the disease diagnosis and treatment guidelines. For some diseases, the guideline usually requires a three-month follow-up, so I will ask the patient to go to the internet hospital for a follow-up visit and prescription. At the third month, I will notify the patient to come to face-to-face. But for IgA nephropathy, the guideline requires patients to have a follow-up once a week, so I will not let IgA nephropathy patients go to the internet hospital for follow-up.

P3, associate chief physician, Nephrology department

Patients use online regular follow-up visit services, with some doing so voluntarily and others being recommended by their doctors. At such times that clinical evaluation or diagnostic testing is warranted, the physician may additionally counsel patients to pursue in-person medical attention. For patients exhibiting superior adherence, the paradigm of online pharmaceutical renewal consultations coupled with periodic in-person evaluations may ensure requisite medical care quality.

For patients in our department, our physicians generally recommend that patients follow up face-to-face once every 3 months. I will regularly post an announcement on the internet hospital platform for patients to follow up face to face once every 3 months. For patients with hepatitis B in our department, 3-monthly follow-up is good for controlling the progression of the disease. If the patient is not followed up at 6 months or even a year, then the situation will be very bad.

P1, associate chief physician, Infectious Diseases department

This study focused on the online regular follow-up visit service. In practice, all patients had previously visited the hospital for an initial face-to-face visit, during which the physician obtained a comprehensive understanding of their medical condition. After this visit, the physician recommended that the patient continue their care with the internet hospital for regular follow-up visits and online prescriptions. This model is likely to improve the quality of the online regular follow-up visit service by ensuring that patients are under the care of a physician who is familiar with their medical history.

The online regular follow-up visit service provided by the THYSYSU internet hospital requires a preliminary offline, face-to-face clinical evaluation. During the initial face-to-face consultation, I will determine the patient's condition through physical examination and some supporting examinations. I will let the patient whose condition is more stable and who is far away have a follow-up consultation online.

P2, associate chief physician, Cardiovascular department

There is communication between the physician and patient during the online regular follow-up visit service. As part of the registration process for internet hospital services, patients are required to provide a comprehensive description of their medical situation. During the online consultation, patients can ask the physician up to 3 questions. The physician will then assess the patient's condition based on the description of the condition and the questions asked. If the patient's condition is stable, the physician may prescribe a prescription online. However, if the patient's condition has changed significantly, the physician will advise the patient to seek in-person care at the hospital. This online communication between physician and patient allows the physician to assess the patient's condition and make treatment recommendations accordingly.

I make my decision based on the patient’s description of their condition. For example, if a patient reports chest tightness and chest pain, I would advise them to go to the hospital immediately. If a patient reports poor blood pressure control during their usual blood pressure monitoring, I would adjust their medication online. The problem is analyzed on a case-by-case basis.

P2, associate chief physician, Cardiovascular department

These practices contribute to the quality of the online regular follow-up visit service. However, according to the interviewees, the effectiveness of the online regular follow-up visit service is still limited compared with face-to-face medical services. They cannot fully replace face-to-face medical services because patients may overlook symptoms that indicate a changing health condition due to their lack of medical expertise.

## Discussion

### Principal Findings

This article synthesizes the supply-side and demand-side dynamics of the TAHSYSU internet hospital together with our quantitative analysis and qualitative interview findings, in order to elucidate the benefits and limitations of online medical facilities with respect to the biological determinant of morbidity patterns as well as the 3 dimensions of health care access, expenditures, and quality. Empirical investigations have shown that the cohort that uses internet hospital services is predominantly composed of patients with chronic diseases with a spatially heterogeneous distribution. Online regular follow-up visit services reduce the time related to registration, waiting for the appointment, and travel, as well as transportation-related costs, but they also increase physician workloads. Physicians leverage patient selection, condition dialogue, and the offline regular follow-up visit service to ensure the quality of online medical care, as virtual services cannot fully replace in-person health care.

The ability of internet hospitals to overcome the constraints of time and space is a widely held view among internet hospital researchers [15,26,33]. However, in our study, the online regular follow-up visit service requires the patients to have a face-to-face consultation first to determine their condition. Therefore, the initial face-to-face consultation does not overcome the time and space constraints, but the subsequent online regular follow-up visit service does. This feature determines that most of the patients using the online regular follow-up visit service come from Guangdong province or provinces closer to Guangdong province. Patients can readily consult clinicians regarding their conditions at any time and in any locale, yet the inability to perform physical examinations or diagnostic tests severely limits clinical determinations based solely on patient narratives. Physicians are not inclined to make a definitive diagnosis and prescription when evaluating patients who have not been seen face to face. It is important to dialectically think about the features that internet hospitals can use to overcome the limitations of time and space.

The type of disease, as a biological factor, influences both the patient’s and physician’s use of internet hospitals. Different diseases require different diagnostic and therapeutic methods, and the characteristics of the disease also determine whether the disease is suitable for online regular follow-up visit services. For clinicians, the decision to provide online regular follow-up visit services depends on the chronicity or acuity of the conditions under their care, as well as the number of patients. Additionally, the income of physicians in different departments affects their willingness to provide online services. These points were confirmed in our interviews with physicians. After excluding patients with a diagnosis of counseling or who had missing diagnosis records, more than one-half of the patients were patients with liver disease. Nearly one-half of the medications on the medication record were antivirals for viral hepatitis. The Infectious Diseases department at TAHSUSY is a national key clinical specialty, and there are a lot of patients going to the Infectious Diseases department. The vast majority of patients who received online regular follow-up visit services were patients with chronic hepatitis. The chronic nature of these diseases allows physicians to reassure patients that they can be followed up online and to serve more patients at the same time. Patients with chronic diseases can also use the online regular follow-up visit service with confidence.

This analysis uses multiple dimensions of accessibility, including appointment, geographic, prescription, and medication availability, to evaluate the online regular follow-up visit service. Specifically, we measured appointment and waiting times, travel time via various transport modes, consultation and prescription times, and medication delivery times. The results of this study show that online regular follow-up visit services have superior appointment and geographic accessibility than in-person consultations. Prescription accessibility is equivalent between online and in-person services, while medication accessibility is inferior with the online regular follow-up visit service. For in-person consultations, patients must book appointments 1 week in advance, without immediate face-to-face access. Although online patients await physicians’ replies postregistration, responses are furnished within 24 hours. Despite proximity, in-person visits entail travel time, whereas online consultations transcend spatiotemporal constraints without travel requirements. Regarding prescription accessibility, physicians expend minutes verifying conditions before prescribing. Consequently, the online regular follow-up visit service confers no advantages over in-person follow-ups in this dimension. However, the online regular follow-up visit service demonstrates inferior medication accessibility, as in-person patients can directly obtain their medications after the consultation and prescribing of medicines, while online patients rely on delivery. In summary, despite deficient medication accessibility, the online regular follow-up visit service demonstrates superior availability overall. Moreover, with proper planning, patients with chronic diseases accessing the online regular follow-up visit service need not urgently require medications.

The cost advantages of the online regular follow-up visit service are more pronounced than the advantages for service accessibility. In addition to the requisite medication and registration expenses, the online regular follow-up visit service saved transportation costs that constitute indirect medical fees. Greater distances from the hospital amplify the economic benefits conferred by internet hospitals. Research undertaken at Huashan Hospital, which is affiliated with Fudan University, used questionnaire instruments to quantify cost and time savings conferred by internet hospitals [34]. We used a new method to estimate the cost and time savings of internet hospitals, which was superior to the questionnaire method. Traditional questionnaire methodology necessitates completion by respondents. Conversely, our novel approach merely requires approximate patient address information. Upon converting addresses into geolocation coordinates and accessing the Amap web service API for vehicular and public transport routing, the time expenditure and travel expenses incurred by patients undergoing in-person clinical visits can be approximated. The predominant virtues of this methodology encompass heightened accuracy compared with traditional questionnaire protocols and mitigation of retrospective bias among the study population. Furthermore, this strategy facilitates analysis of indirect medical costs.

### Comparison With Prior Work

According to Donabedian [35], quality can be defined as “structure-process-outcome” quality. Qi et al [34] and Chen et al [36] partially assessed the medical quality of internet hospitals by administering patient satisfaction surveys regarding their use of online services. Patient satisfaction can be an indicator of outcome quality. In our research, the quantitative research mainly focused on the process quality of online health care. In our opinion, the control of the quality of medical care in the medical process is more important than the satisfaction with the demand side. The difference in medical knowledge between physicians and patients is enormous. Patients’ perceptions of the quality of medical care are relatively subjective, and the provider is the controller of the quality of care in the health service process. According to our quantitative research, patients visit the doctor an average of more than 2 times, with some patients even visiting 6 times. Patients’ repeated use of the online regular follow-up visit service indicates their recognition of the quality of care provided by the internet hospital. According to our qualitative research, physicians mainly understand the patient's condition during the initial consultation. During the online regular follow-up visit service, they understand the patient’s disease status based on the patient’s description and communication between the 2 parties. By comparing the patient’s condition during the initial consultation and communication during the regular follow-up visit, physicians can judge whether the effect of the patient's online follow-up consultation is similar to that of the face-to-face consultation. These measures help physicians ensure the quality of care during the online follow-up.

### Limitations

This article has several limitations. First, as a single-center study in 1 sample hospital, it may have biases in the research results. Although a focused hospital study can provide detailed descriptions, there are significant regional, urban-rural, and typological differences among China’s public hospitals. Thus, the findings from this comprehensive hospital in a southeastern coastal region may not reflect the situation of public hospitals in underdeveloped areas in China. Wider collaboration with multiple hospitals in a representative, nationwide, multicenter study is needed in the future. Second, perspectives from the demand side—patients receiving online health care—regarding its quality are lacking. In the next phase, more detailed analysis on online health care quality from both demand and supply sides will be conducted.

### Conclusions

Telemedicine services such as online regular follow-up visit services provided by internet hospitals must also strictly adhere to fundamental medical principles of diagnosis, prescription, and treatment. Therefore, the ability of internet hospitals to overcome the constraints of time and space depend upon the principles of disease diagnosis. The initial face-to-face consultation does not overcome the time and space constraints because of testing, but the subsequent regular online follow-up visit service does. Different diseases necessitate different diagnostic and therapeutic approaches. For chronic illnesses or chronic phases of disease, the patient's condition often changes relatively little over time. These stable cases with minimal fluctuation are well-suited for regular online follow-up visit services. Overall, for patients with chronic diseases, internet hospitals demonstrate significant advantages, both in accessibility and cost. Although physicians utilize various methods to ensure the quality of online health care, the online regular follow-up visit service cannot fully replace in-person medical services.

## Appendix

Multimedia Appendix 1 Distribution of patients across geographies and hospital departments.

Multimedia Appendix 2 Interview transcripts.

## Abbreviations

API: application programming interface
TAHSYSU: Third Affiliated Hospital of Sun Yat-sen University
WHO: World Health Organization

## References

[1] Sood S, Mbarika V, Jugoo S, Dookhy R, Doarn CR, Prakash N, Merrell RC (2007). What is telemedicine? A collection of 104 peer-reviewed perspectives and theoretical underpinnings. Telemed J E Health.

[2] Jagarapu J, Savani RC (2021). A brief history of telemedicine and the evolution of teleneonatology. Semin Perinatol.

[3] Riva G, Gamberini L (2000). Virtual reality in telemedicine. Telemed J E Health.

[4] Jiang F, Jiang Y, Zhi H, Dong Y, Li H, Ma S, Wang Y, Dong Q, Shen H, Wang Y (2017). Artificial intelligence in healthcare: past, present and future. Stroke Vasc Neurol.

[5] Tian W (2020). [Exploration and prospect of 5G application in telemedicine]. Zhonghua Wai Ke Za Zhi.

[6] Rowan C, Hirten R (2022). The future of telemedicine and wearable technology in IBD. Curr Opin Gastroenterol.

[7] Zheng J, Wang Y, Zhang J, Guo W, Yang X, Luo L, Jiao W, Hu X, Yu Z, Wang C, Zhu L, Yang Z, Zhang M, Xie F, Jia Y, Li B, Li Z, Dong Q, Niu H (2020). 5G ultra-remote robot-assisted laparoscopic surgery in China. Surg Endosc.

[8] Jiang X (2017). Reshaping the healthcare landscape: a brief history of China's mobile healthcare development. Internet Economy.

[9] (2018). Circular of the Chinese Medicine Bureau on the Issuance of Three Documents, Including the Measures for the Administration of Internet Diagnosis and Treatment (for Trial Implementation). National Health and Medical Administration.

[10] Kitamura C, Zurawel-Balaura L, Wong RKS (2010). How effective is video consultation in clinical oncology? A systematic review. Curr Oncol.

[11] Hong Z, Li N, Li D, Li J, Li B, Xiong W, Lu L, Li W, Zhou D (2020). Telemedicine during the COVID-19 pandemic: experiences from western China. J Med Internet Res.

[12] Anand S, Fan VY, Zhang J, Zhang L, Ke Y, Dong Z, Chen LC (2008). China's human resources for health: quantity, quality, and distribution. The Lancet.

[13] Wan S, Chen Y, Xiao Y, Zhao Q, Li M, Wu S (2021). Spatial analysis and evaluation of medical resource allocation in China based on geographic big data. BMC Health Serv Res.

[14] Wang T, Li J, Zhu C, Hong Z, An D, Yang H, Ren J, Zou X, Huang C, Chi X, Chen J, Hong Z, Wang W, Xu C, He L, Li W, Zhou D (2016). Assessment of utilization and cost-effectiveness of telemedicine program in western regions of China: a 12-year study of 249 hospitals across 112 cities. Telemed J E Health.

[15] Tu J, Wang C, Wu S (2015). The internet hospital: an emerging innovation in China. Lancet Glob Health.

[16] Han Y, Lie RK, Guo R (2020). The internet hospital as a telehealth model in China: systematic search and content analysis. J Med Internet Res.

[17] The Second People's Hospital of Guangdong Province (2016). Sending Experts to the Village Entrance: Internet Hospitals.

[18] (2018). Opinions of the General Office of the State Council on Promoting the Development of "Internet+Medical Health". General Office of the State Council.

[19] Xie X, Zhou W, Lin L, Fan S, Lin F, Wang L, Guo T, Ma C, Zhang J, He Y, Chen Y (2017). Internet hospitals in China: cross-sectional survey. J Med Internet Res.

[20] (2019). National Health Commission: the country now has 158 Internet hospitals. Xinhua News Agency.

[21] Xiaohang T (2021). Rapid development! China's Internet hospitals have reached more than 1,600. Xinhua News Agency.

[22] Wang W, Sun L, Liu T, Lai T (2022). The use of E-health during the COVID-19 pandemic: a case study in China's Hubei province. Health Sociol Rev.

[23] Sun S, Yu K, Xie Z, Pan X (2020). China empowers Internet hospital to fight against COVID-19. J Infect.

[24] (2021). There are 7,700 hospitals above the second level in the country providing online services Internet healthcare, making patients easier to see a doctor. People's Daily.

[25] Ding L, She Q, Chen F, Chen Z, Jiang M, Huang H, Li Y, Liao C (2020). The internet hospital plus drug delivery platform for health management during the COVID-19 pandemic: observational study. J Med Internet Res.

[26] Gong K, Xu Z, Cai Z, Chen Y, Wang Z (2020). Internet hospitals help prevent and control the epidemic of COVID-19 in China: multicenter user profiling study. J Med Internet Res.

[27] Xu L, Yin X, Geller J, Li Y, Zhou R, Wang H, Zhang Y (2016). Internet Hospital: Challenges and Opportunities in China. Health Information Science. HIS 2016. Lecture Notes in Computer Science(), vol 10038.

[28] Ge F, Qian H, Lei J, Ni Y, Li Q, Wang S, Ding K (2022). Experiences and challenges of emerging online health services combating COVID-19 in China: retrospective, cross-sectional study of internet hospitals. JMIR Med Inform.

[29] Li Y, Hu H, Rozanova L, Fabre G (2022). COVID-19 and internet hospital development in China. Epidemiologia (Basel).

[30] (2010). Monitoring the building blocks of health systems: a handbook of indicators and their measurement strategies. World Health Organization.

[31] Andersen RM, Davidson PL, Andersen RM, Rice TH, Kominski GF (2007). Improving Access to Care in America: Individual and Contextual Indicators. Changing the U.S. health care system: Key issues in health services policy and management.

[32] (2000). The world health report 2000. World Health Organization.

[33] Xu X, Cai Y, Wu S, Guo J, Yang L, Lan J, Sun Y, Wang B, Wu J, Wang T, Huang S, Lin Y, Hu Y, Chen M, Gao X, Xie X (2021). Assessment of internet hospitals in China during the COVID-19 pandemic: national cross-sectional data analysis study. J Med Internet Res.

[34] Qi H, Ying Y, Zhu L, Li Q, Wang T, Chen B, Zhong M (2023). Exploration on the development of public hospital-sponsored telemedicine platform: A case study in China. J Telemed Telecare.

[35] Donabedian A (1966). Evaluating the quality of medical care. Milbank Mem Fund Q.

[36] Chen Z, Liang W, Liu Q, He R, Chen Q, Li Q, Zhang Y, Du X, Pan Y, Liu S, Li X, Wei X, Huang H, Huang H, Liu T (2021). Use of a remote oncology pharmacy service platform for patients with cancer during the COVID-19 pandemic: implementation and user acceptance evaluation. J Med Internet Res.

