# Supplementary material for: Accessibility, Cost, and Quality of an Online Regular Follow-Up Visit Service at an Internet Hospital in China: Mixed Methods Study
Source: J Med Internet Res. 2024 Oct 21;26:e54902. doi: 10.2196/54902 (PMC11535792; doi:10.2196/54902)
Supplement: Multimedia Appendix 1 [file jmir_v26i1e54902_app1.docx]

Table S1. Province-level geographical distribution of patients and average number of visits in China

| **Provinces** | **Number of Visits** | **Proportion**  **(%)** | **Number of Patients** | **Proportion**  **(%)** | **Average Visits per person** |
| --- | --- | --- | --- | --- | --- |
| Guangdong | 36310 | 92.54% | 16979 | 91.91% | 2.14 |
| Hunan | 545 | 1.39% | 277 | 1.50% | 1.97 |
| Jiangxi | 497 | 1.27% | 243 | 1.32% | 2.05 |
| Hainan | 429 | 1.09% | 181 | 0.98% | 2.37 |
| Guangxi | 424 | 1.08% | 216 | 1.17% | 1.96 |
| Hubei | 135 | 0.34% | 83 | 0.45% | 1.63 |
| Henan | 115 | 0.29% | 66 | 0.36% | 1.74 |
| Zhejiang | 88 | 0.22% | 52 | 0.28% | 1.69 |
| Guizhou | 88 | 0.22% | 34 | 0.18% | 2.59 |
| Fujian | 86 | 0.22% | 51 | 0.28% | 1.69 |
| Sichuan | 80 | 0.20% | 44 | 0.24% | 1.82 |
| Yunnan | 62 | 0.16% | 29 | 0.16% | 2.14 |
| Jiangsu | 48 | 0.12% | 27 | 0.15% | 1.78 |
| Anhui | 48 | 0.12% | 24 | 0.13% | 2.00 |
| Shandong | 43 | 0.11% | 21 | 0.11% | 2.05 |
| Beijing | 36 | 0.09% | 20 | 0.11% | 1.80 |
| Shanxi | 32 | 0.08% | 14 | 0.08% | 2.29 |
| Chongqing | 30 | 0.08% | 21 | 0.11% | 1.43 |
| Shanghai | 30 | 0.08% | 19 | 0.10% | 1.58 |
| Liaoning | 18 | 0.05% | 10 | 0.05% | 1.80 |
| Xinjiang | 16 | 0.04% | 6 | 0.03% | 2.67 |
| Shaanxi | 16 | 0.04% | 10 | 0.05% | 1.60 |
| Heilongjiang | 16 | 0.04% | 11 | 0.06% | 1.45 |
| Hebei | 13 | 0.03% | 10 | 0.05% | 1.30 |
| Qinghai | 11 | 0.03% | 6 | 0.03% | 1.83 |
| Tianjin | 9 | 0.02% | 6 | 0.03% | 1.50 |
| Inner Mongolia | 6 | 0.02% | 5 | 0.03% | 1.20 |
| Jilin | 4 | 0.01% | 4 | 0.02% | 1.00 |
| Gansu | 4 | 0.01% | 4 | 0.02% | 1.00 |
| **Total** | **39239** | **100%** | **18473** | **100%** | **2.12** |

Note: If two patients have same district and id information, they will be recognized as the same person.

Table S2 City-level geographical distributions of patients and average number of visits in Guangdong Province

| **Cities** | **Number of Visits** | **Proportion**  **(%)** | **Number of Patients** | **Proportion**  **(%)** | **Average Visits per person** |
| --- | --- | --- | --- | --- | --- |
| Guangzhou | 15903 | 43.80% | 8004 | 47.14% | 1.99 |
| Heyuan | 1216 | 3.35% | 515 | 3.03% | 2.36 |
| ZhaoQing | 1621 | 4.46% | 702 | 4.13% | 2.31 |
| Yunfu | 850 | 2.34% | 345 | 2.03% | 2.46 |
| Shanwei | 797 | 2.19% | 378 | 2.23% | 2.11 |
| Qingyuan | 1241 | 3.42% | 543 | 3.20% | 2.29 |
| Foshan | 2412 | 6.64% | 1131 | 6.66% | 2.13 |
| Yangjiangy | 692 | 1.91% | 297 | 1.75% | 2.33 |
| Gongguan | 2799 | 7.71% | 1180 | 6.95% | 2.37 |
| Huizhou | 1491 | 4.11% | 637 | 3.75% | 2.34 |
| Jieyang | 1017 | 2.80% | 427 | 2.51% | 2.38 |
| Jiangmen | 933 | 2.57% | 367 | 2.16% | 2.54 |
| Chaozhou | 396 | 1.09% | 175 | 1.03% | 2.26 |
| Shaoguan | 435 | 1.20% | 177 | 1.04% | 2.46 |
| Maoming | 865 | 2.38% | 369 | 2.17% | 2.34 |
| Zhongshan | 542 | 1.49% | 255 | 1.50% | 2.13 |
| Zhanjiang | 899 | 2.48% | 400 | 2.36% | 2.25 |
| Zhuhai | 249 | 0.69% | 123 | 0.72% | 2.02 |
| Meizhou | 392 | 1.08% | 181 | 1.07% | 2.17 |
| Shantou | 555 | 1.53% | 256 | 1.51% | 2.17 |
| Shenzhen | 1005 | 2.77% | 517 | 3.04% | 1.94 |
| **Total** | **36310** | **100.00%** | **16979** | **100%** | **2.14** |

Note: If two patients have same district and id information, they will be recognized as the same person.

Table S3. District-level geographical distributions of patients and average number of visits in Guangzhou City

| **Districts** | **Number of Visits** | **Proportion**  **(%)** | **Number of Patients** | **Proportion**  **(%)** | **Average Visits per person** |
| --- | --- | --- | --- | --- | --- |
| Huangpu | 3267 | 20.54% | 1470 | 18.37% | 2.22 |
| Tianhe | 3845 | 24.18% | 2166 | 27.06% | 1.78 |
| Zengcheng | 1752 | 11.02% | 800 | 10.00% | 2.19 |
| Panyu | 2152 | 13.53% | 1075 | 13.43% | 2.00 |
| Yuexiu | 537 | 3.38% | 295 | 3.69% | 1.82 |
| Haizhu | 923 | 5.80% | 507 | 6.33% | 1.82 |
| Nansha | 478 | 3.01% | 212 | 2.65% | 2.25 |
| Liwan | 491 | 3.09% | 246 | 3.07% | 2.00 |
| Baiyun | 1492 | 9.38% | 802 | 10.02% | 1.86 |
| Conghua | 303 | 1.91% | 140 | 1.75% | 2.16 |
| Huadu | 663 | 4.17% | 291 | 3.64% | 2.28 |
| **Total** | **15903** | **100%** | **8004** | **100%** | **1.99** |

Note: If two patients have same district and id information, they will be recognized as the same person.

Table S4. The distribution of patients across hospital departments and average visits per person

| **Department** | **Number of Visits** | **Proportion**  **(%)** | **Number of Patients** | **Proportion**  **(%)** | **Average Visits per person** |
| --- | --- | --- | --- | --- | --- |
| Infectious Diseases | 26163 | 66.68 | 10935 | 59.2 | 2.39 |
| Rheumatology and Immunology | 2804 | 7.15 | 1442 | 7.81 | 1.94 |
| Dermatology | 2643 | 6.74 | 1828 | 9.9 | 1.45 |
| Gynecology | 1122 | 2.86 | 732 | 3.96 | 1.53 |
| Hepatobiliary Surgery | 941 | 2.4 | 356 | 1.93 | 2.64 |
| Endocrinology | 857 | 2.18 | 522 | 2.83 | 1.64 |
| Neurology | 714 | 1.82 | 315 | 1.71 | 2.27 |
| Cardiovascular Internal Medicine | 670 | 1.71 | 378 | 2.05 | 1.77 |
| Hepatobiliary Surgery | 565 | 1.44 | 295 | 1.6 | 1.92 |
| Nephrology | 533 | 1.36 | 273 | 1.48 | 1.95 |
| Gastroenterology | 395 | 1.01 | 209 | 1.13 | 1.89 |
| Traditional Chinese Medicine | 193 | 0.49 | 105 | 0.57 | 1.84 |
| Interventional Radiology | 184 | 0.47 | 83 | 0.45 | 2.22 |
| Respiratory Internal Medicine | 151 | 0.38 | 100 | 0.54 | 1.51 |
| Urology | 143 | 0.36 | 91 | 0.49 | 1.57 |
| Obstetrics | 136 | 0.35 | 99 | 0.54 | 1.37 |
| Breast and Mammary Surgery | 89 | 0.23 | 66 | 0.36 | 1.35 |
| Otolaryngology (Ear, Nose, and Throat) | 86 | 0.22 | 55 | 0.3 | 1.56 |
| Reproductive Medicine and Andrology | 83 | 0.21 | 54 | 0.29 | 1.54 |
| Kidney Transplantation | 81 | 0.21 | 42 | 0.23 | 1.93 |
| Ophthalmology | 78 | 0.2 | 58 | 0.31 | 1.34 |
| Spinal Surgery | 75 | 0.19 | 55 | 0.3 | 1.36 |
| Psychiatry | 74 | 0.19 | 67 | 0.36 | 1.1 |
| Hematology | 70 | 0.18 | 35 | 0.19 | 2 |
| Ophthalmology | 65 | 0.17 | 50 | 0.27 | 1.3 |
| General Medicine | 56 | 0.14 | 34 | 0.18 | 1.65 |
| Reproductive Medicine Center | 45 | 0.11 | 33 | 0.18 | 1.36 |
| Joint Surgery | 36 | 0.09 | 33 | 0.18 | 1.09 |
| Neurosurgery | 34 | 0.09 | 18 | 0.1 | 1.89 |
| Otolaryngology (Ear, Nose, and Throat) | 32 | 0.08 | 25 | 0.14 | 1.28 |
| Orthopedics | 26 | 0.07 | 21 | 0.11 | 1.24 |
| Allergy and Immunology | 24 | 0.06 | 16 | 0.09 | 1.5 |
| Respiratory and Critical Care Medicine | 23 | 0.06 | 16 | 0.09 | 1.44 |
| Ultrasound Department | 19 | 0.05 | 12 | 0.06 | 1.58 |
| Medical Oncology | 12 | 0.03 | 6 | 0.03 | 2 |
| Pain Management | 5 | 0.01 | 5 | 0.03 | 1 |
| Rehabilitation Medicine | 5 | 0.01 | 3 | 0.02 | 1.67 |
| Cerebrovascular Surgery | 3 | 0.01 | 3 | 0.02 | 1 |
| Anorectal Surgery | 2 | 0.01 | 2 | 0.01 | 1 |
| Vascular Surgery | 2 | 0.01 | 1 | 0.01 | 2 |
| **Total** | **39239** | **100** | **18473** | **100** | **2.12** |

Note: If two patients have same district and id information, they will be recognized as the same person.
