# Supplementary material for: Accessibility, Cost, and Quality of an Online Regular Follow-Up Visit Service at an Internet Hospital in China: Mixed Methods Study
Source: J Med Internet Res. 2024 Oct 21;26:e54902. doi: 10.2196/54902 (PMC11535792; doi:10.2196/54902)
Supplement: Multimedia Appendix 2 [file jmir_v26i1e54902_app2.docx]

Table 1 Interviewee's department, title, and symbol number

| **Department** | **Technical titles** | **Symbol number** |
| --- | --- | --- |
| Department of Infectious diseases | Associate chief physician | P1 |
| Department of Cardiovascular | Associate chief physician | P2 |
| Department of Nephrology | Associate chief physician | P3 |
| Department of Endocrinology | Associate chief physician | P4 |
| Department of Psychiatry | Chief physician | P5 |
| Department of Psychiatry | Chief physician | P6 |
| Department of Infectious diseases | Chief physician | P7 |
| Department of Outpatient office | Chief physician | P8 |
| Department of Outpatient office | Section Member | P9 |
| Department of Outpatient office | Section Member | P10 |
| Department of Outpatient office | Head nurse | P11 |
| Department of Outpatient office | Head nurse | P12 |

**Interview transcripts used in this paper**

Because we used semi-structured interviews, we distilled the respondents' clearest answers to the questions and translated them into English. The content of the interviews with the three doctors P5 to P7 focused on the history of internet healthcare, willingness for online diagnosis and treatment. The three Outpatient Department interviewees were informed about the development and management of Internet hospitals, while the two interviewed Outpatient Department head nurses were asked about the nurses' attitudes and support for internet healthcare. Hence, this information was not included in this article.

**P1, Associate chief physician in Department of Infectious diseases**

1) How to ensure the medical service quality during the online regular follow-up visit service process?

For patients in our department, our physicians generally recommend that patients follow up face-to-face once every 3 months. I will regularly post an announcement on the Internet hospital platform for patients to follow up face-to-face once every 3 months. For patients with hepatitis B in our department, 3-monthly follow-up is good for controlling the progression of the disease. If the patient is not followed up at 6 months or even a year, then the situation will be very bad. One of the good features of the Internet hospital is that it can send out an announcement that we recommend patients to come for follow-up in 3 months, and if they can't come for other reasons, I will also send out an announcement to recommend patients to come for follow-up near them, and if liver cancer is detected within the 3-month period, it is also in the early stage, and the prognosis is relatively optimistic compared to advanced liver cancer.

2) Which diseases and conditions would you recommend for patients to have online regular follow-up visit service?

I believe that the diseases that can be revisited online are related to the characteristics of the disease itself, which can be divided into acute and chronic diseases, and in our department most of the patients with hepatitis B are revisited online, which is a chronic infectious disease. In this case, the patient's need is to receive medical care in a consistent manner. For hepatitis B patients, most of them need to take antiviral drugs for life, so they need to go to the hospital for a long time to prescribe medication, and it is not realistic for them to come to the hospital every time to prescribe medication, so the Internet hospital has developed in this way.

3) Is there any difference between the types of drugs prescribed online and those prescribed offline?

We prescribe the same drugs to both online and face-to-face patients, because of the current health insurance policy, basically all drugs prescribed in hospitals are centralized nationally procured drugs.

4) What other perspectives do you have on the quality of healthcare services provided by online hospitals?

Overly convenient online follow-up consultations may lead to faster progression of the patient's disease and a poor prognosis of the disease. Every patient has a certain degree of inertia, and with the convenience of the Internet hospital follow-up consultation and medication sending service, some patients may over-rely on online follow-up consultation and neglect the progress of their disease. Some patients think that it is enough to have medication, but due to the drug resistance of hepatitis B virus and the blockade and isolation of the epidemic, these patients come to the hospital for offline follow-up once in a long time, and as a result, liver cancer is detected, and the disease can not be controlled by medication alone.

5) What else do you think about online regular follow-up visit service?

In a traditional face-to-face consultation, the doctor can spend 5-10 minutes to fully understand the patient's condition. I have participated in a conference on Internet healthcare before, and many very famous experts and practitioners of Internet hospitals have indicated that Internet hospitals cannot completely replace traditional hospitals, but just from the level of online medication refills for chronically ill patients, Internet hospitals have no problem at all. Our hospital is the patient initiated online follow-up request, the doctor will communicate with the patient within 24 hours, sometimes, the doctor is too busy to deal with online follow-up patients. When they ended their job, the patient is not online, there is a time difference between the two sides, which indirectly leads to the communication between doctors and patients is inefficient. And the epidemic has a greater impact on the regular follow-up of patients, and Internet hospitals develop faster during epidemics.

**P2, Associate chief physician in Department of Cardiovascular**

1) How to ensure the medical service quality during the online regular follow-up visit service process?

The online regular follow-up visit service provided by the THYSYSU internet hospital require a preliminary offline, face-to-face clinical evaluation. During the initial face-to-face consultation, I will determine the patient's condition through physical examination and some supporting examinations. I will let the patient whose condition is more stable and who is far away to have a follow-up consultation online.

I make my decision based on the patient's description of their condition. For example, if a patient reports chest tightness and chest pain, I would advise them to go to the hospital immediately. If a patient reports poor blood pressure control during their usual blood pressure monitoring, I would adjust their medication online. The problem is analyzed on a case-by-case basis.

2) Which diseases and conditions would you recommend for patients to have online regular follow-up visit service?

The first is patients who have been seen face-to-face, such as those whose condition was judged to be stable during the initial face-to-face consultation and whose medication is relatively stable. Secondly, I would recommend online follow-up for patients who have undergone complementary tests at the initial visit and I am more certain that their condition will be stabilized under the control of medication. Lastly, for those patients whose subjective feelings do not change much during the online follow-up, I will recommend them to continue the online follow-up.

3) Is there any difference between the types of drugs prescribed online and those prescribed offline?

I have never prescribed medication to a patient at an Internet hospital on a third-party platform such as Haodaifu. But on the TAHSYSU Internet platform, sometimes I can't stand the patient's request to prescribe drugs to the first-time patients. Usually, the drugs I prescribe are safer and more conservative, and I will not prescribe drugs with stronger side effects to my patients.

4) What other perspectives do you have on the quality of healthcare services provided by online hospitals?

I think our hospital's quality assurance for online treatment is superior to that of Internet hospitals on other platforms, because brick-and-mortar hospital-led Internet hospitals, after all, have an official nature in them. And patients who choose to follow up online are those who must have had their initial visit offline.

5) What else do you think about online regular follow-up visit service?

I think a big reason why many doctors do not provide online services is due to safety issues, even on the hospital's Internet platform, and they are concerned about the negative impact on the patient due to the lack of understanding of the patient's condition in the online revisit. However, for us internists, providing online regular follow-up services for stabilized patients can be beneficial in saving time for both parties, and can also generate an income.

**P3, Associate chief physician in Department of Nephrology**

1) How to ensure the medical service quality during the online regular follow-up visit service process?

I will decide whether the patient will have an online follow-up or a face-to-face follow-up based on the disease diagnosis and treatment guidelines. For some diseases, the guideline usually requires a three-month follow-up, so I will ask the patient to go to the Internet hospital for a follow-up visit and prescription. At the third month, I will notify the patient to come to face-to-face. But for IgA nephropathy, the guideline requires patients to have a follow-up once a week, so I will not let IgA nephropathy patients go to the Internet hospital for follow-up.

The patients for whom I offer online regular follow-up services are those who have already received an initial consultation with me face-to-face. After the face-to-face consultation, based on the patient's condition, I determine that these patients need to come in for a follow-up consultation every three months. Therefore, I agreed with them that this time I will prescribe you medication for one month. Next month, you will come directly to the Internet hospital to online follow-up. I will prescribe you drugs through the Internet hospital.

2) Which diseases and conditions would you recommend for patients to have online regular follow-up visit service?

I would recommend that after the initial face-to-face diagnosis, in accordance with the guidelines for the treatment of the diagnosed disease, patients whose diagnosis is clear, whose treatment plan is clear, whose medication is clear, and whose condition is stabilized should come to the online follow-up consultation.

3) Is there any difference between the types of drugs prescribed online and those prescribed offline?

They are all drugs that have gone through centralized national procurement with volume purchasing

4) What other perspectives do you have on the quality of healthcare services provided by online hospitals?

Generally speaking, medication refills are better suited to be done online because the patient is judged to be stable after a face-to-face consultation, but I do not usually diagnose patients online because the online consultation process is prone to omissions that may result in an inaccurate judgment of the patient's condition.

5) What else do you think about online regular follow-up visit service?

At present, there are still a lot of inconvenient places in the process of online diagnosis and treatment, face-to-face diagnosis and treatment process can always retrieve the patient's previous diagnosis and treatment results and records for comparison. In the process of online diagnosis and treatment, the process of comparison is more troublesome.

**P4, Associate chief physician in Department of Endocrinology**

1) How to ensure the medical service quality during the online regular follow-up visit service process?

In our endocrinology department, the patients I usually deal with most are patients with diabetes and hyperthyroidism. For patients with these two diseases, doctors need to adjust the treatment plan according to the patient's daily situation. We need to ask diabetic patients about complications during online consultations and ask them to come to the hospital for face-to-face follow-ups if they are experiencing related symptoms. For patients with hyperthyroidism, our doctors need to ask about the side effects of the medication. For some young women with hyperthyroidism, we also ask if they are planning to get pregnant. The decision as to whether to follow up online or offline is made on a case-by-case basis.

2) Which diseases and conditions would you recommend for patients to have online regular follow-up visit service?

Usually, I would recommend that patients who have had their first face-to-face consultation come to the Internet hospital to get their medication online, but the patients in our department are usually elderly patients with chronic diseases, so I would not recommend that the elderly go to the Internet hospital for follow-up consultations and prescriptions, as it may be difficult for them to operate their cell phones. However, I would recommend that the younger members of their families help them to do so. For patients with complete medical information and simpler conditions, I would recommend them to go to the Internet hospital for follow-up consultations.

3) Is there any difference between the types of drugs prescribed online and those prescribed offline?

Medical insurance can reimburse most of the daily medications for patients with chronic diseases, and basically there is no difference between the medications for online and offline follow-up patients with the same condition.

4) What other perspectives do you have on the quality of healthcare services provided by online hospitals?

Because the Internet hospital online follow-up service only requires the patient to pay 10 yuan, this price is very friendly to the patient, and the patient does not have to come to the hospital, so this improves the continuity of the patient's follow-up, but also helps us to manage the patient.

5) What else do you think about online regular follow-up visit service?

Internet hospitals are acting as a triage. Continuity of medical care for patients can be enhanced through online regular follow-up visit service at Internet hospitals. But with the increase in the number of patients, we are also struggling. Many patients with stabilized conditions ask a lot of questions during online regular follow-up visit service out of concern for their condition, which increases our workload.
